# Sex-dependent developmental changes in behavior, brain structure, functional connectivity, and sensory perception following exposure to psilocybin during adolescence

**DOI:** 10.1038/s41386-026-02356-8

**Published:** 2026-02-18

**Authors:** Itishree Sahoo, Sairam Masadi, Ashwath Maheswari, Rachel Utama, Muhammad I. Abeer, Sima Soltanpour, Md Taufiq Nasseef, Tochi Chukwuemeka, Nandini Sinhal, Jyot Pandit, Richard J. Ortiz, Noah Cavallaro, Eric Brengel, Praveen P. Kulkarni, Michael A. Gitcho, Craig F. Ferris

**Affiliations:** 1 Center for Translational NeuroImaging, Northeastern Univ, Boston, MA USA; 2 Department of Biological Sciences, Delaware State University, Dover, DE USA; 3 School of Information Technology, Carleton University, Ottawa, ON Canada; 4 Department of Mathematics, College of Science and Humanity Studies, Prince Sattam Bin Abdulaziz University, Riyadh, Saudi Arabia; 5 Department of Psychology, Northern Illinois Univ, DeKalb, IL USA; 6 Depts of Psychology and Pharmaceutical Sciences Northeastern Univ, Boston, MA USA

**Keywords:** Neuronal development, Preclinical research

## Abstract

Psilocybin is a hallucinogen with complex neurobiological and behavioral effects. Underlying these effects are changes in brain neuroplasticity. We hypothesized psilocybin given during adolescence, a time of heightened neuroplasticity, particularly in the forebrain, would affect emotional behavior and the associated underlying neuroanatomy, neurocircuitry, and epigenetics. Female and male mice were given vehicle or 3.0 mg/kg psilocybin every other day by oral gavage from postnatal days 40–50 for a total of five exposures. Between postnatal days 90–120 mice were imaged and evaluated for affective behavior and perception of rewarding and aversive stimuli. MRI data from voxel-based morphometry, diffusion weighted imaging, and BOLD resting state functional connectivity were registered to a mouse 3D MRI atlas with 139 brain regions providing site-specific differences in global brain structure and functional connectivity between experimental groups. The prefrontal cortex was measured for changes in proteins associated with epigenetics. Mice showed no significant differences in the light/dark box test, but female mice exposed to psilocybin showed reduced mobility in the open field as compared to controls. Mice with early psilocybin exposure showed reduced brain sensitivity to both rewarding and aversive odors during scanning sessions. There were regional reductions in brain volume and alteration in water diffusivity affecting males more than females. Global and regional functional connectivity were increased in both sexes with the prefrontal cortex showing enhanced connections to the hypothalamus, thalamus and midbrain. Males showed reduced levels of epigenetic and neuroplasticity protein markers in the prefrontal cortex. The pronounced changes in brain volume, water diffusivity - a surrogate marker of gray matter microarchitecture, increase in functional connectivity, altered perception of rewarding and aversive stimuli and altered levels of protein markers of neuroplasticity provide compelling evidence that exposure to psilocybin during adolescence has long term developmental consequences, particularly in males.

## Introduction

Psilocybin (PSI) has emerged as a compound of significant interest in contemporary psychological research due to its therapeutic potential for treatment-resistant mood and anxiety disorders. While indigenous populations have utilized PSI-containing substances for millennia, scientific investigation was substantially interrupted for approximately three decades following the Drug Enforcement Administration’s classification of psychedelic compounds as Schedule I substances in 1970 [1]. The development of advanced neuroimaging technologies, particularly magnetic resonance imaging (MRI) and positron emission tomography (PET), during the 1990s enhanced scientific understanding of central nervous system mechanisms underlying psychedelic effects. These technological advances catalyzed renewed research interest in psychedelic compounds, ultimately leading to controlled pilot investigations and subsequent Food and Drug Administration approvals for therapeutic applications in treating various psychiatric disorders [2]. Contemporary clinical studies report therapeutic efficacy across multiple domains, including substance use disorders [3], major depressive disorder [1, 4], and anxiety disorders [5].

As PSI-containing mushrooms become increasingly prevalent in contemporary drug culture, there are growing concerns regarding adolescent use during this critical period of neurodevelopment [6, 7]. The 2021 National Survey on Drug Use and Health reported lifetime use of PSI to be almost 10% for 12 year old respondents [8]. In a just published 10-year study, over 4000 PSI-involved exposures were reported among adolescents and young adults [9]. In 2022, cases more than tripled among adolescents as compared to previous years. In a prospective study investigating acute and longer-term psychological effects of psychedelics in adolescence, there was a reported significant increase in psychological well-being in the weeks after psychedelic use [10]. However, there were some noted concerns around ego-dissolution and a higher prevalence of hallucinogen persisting perceptual disorder. Issues around the safety and efficacy of psychedelics in treating adolescent mental illness was addresses in a recent paper by Jaffery and colleagues [11].

Interestingly, there are few studies looking at the effects of PSI in periadolescent animals. Mice ca 45–50 days of age given the hallucinogenic mushroom *P argentipes* show a significant reduction in marble burying a measure of anxiolytic activity [12]. The *P argentipes* mushroom contains a host of psychoactive tryptamine derivative including PSI [13]. However, treatment with PSI alone in this model has very little effect. In a just published study Garcia-Cabreizo et al., tested the antidepressant potential of PSI in ca 35–45 days old rats using the forced swim test [14]. Psilocybin produced a rapid response reducing the time of immobility in both males and females.

The current investigation employed MRI to assess neurodevelopmental alterations in brain structure and function among male and female mice following oral PSI administration during adolescence. Preclinical MRI represents a non-invasive approach for characterizing drug-induced changes in brain function and connectivity throughout development in response to centrally active compounds [15–18]. Although extensive data have been obtained through histological examinations and behavioral assessments in animal models, comprehensive understanding of the region-specific functional impacts of compounds like PSI on neural networks remains limited. This knowledge gap is further compounded by the scarcity of neuroimaging studies conducted in awake compared to anesthetized rodent preparations. Importantly, to our knowledge, no existing preclinical or clinical research has investigated the persistent neurodevelopmental consequences of PSI exposure occurring during the periadolescent developmental window.

## Methods and methods

### Animals

**Fig. 1:**
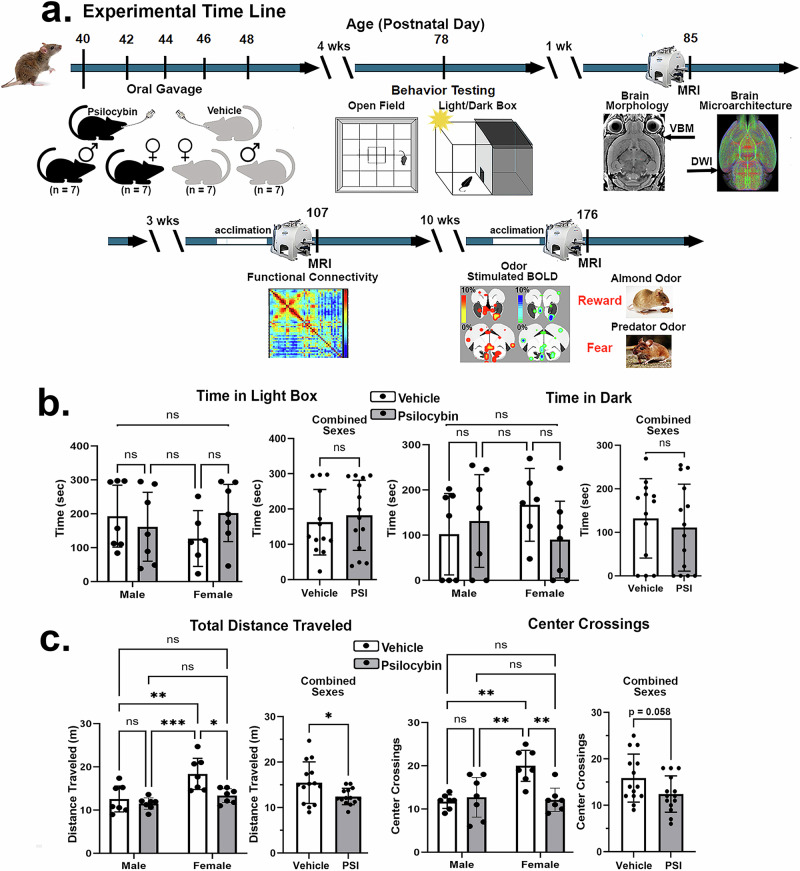
Timeline and behavior. Shown in Fig. 1**a** is the experimental timeline treatment, behavioral testing, and imaging. Below are bar graphs summarizing the results from the light/dark box Fig 1**b** and Open field Fig 1**c**. There were no significant sex differences in light/dark performance. Vehicle treated females were significantly more active than males while PSI treated significantly reduced this behavior. *p < 0.05; **p < 0.01; ***p < 0.001; ****p < 0.0001.

Twenty-eight C57BL/J6 mice, half male/half female, 36 days of age were obtained from Charles River Laboratories (Wilmington, Massachusetts, USA). Animals were housed in plastic cages (3–5 per cage) and maintained in ambient temperature (22–24 °C) on a 12:12 reverse light:dark cycle (lights off at 09:00 h). All experiments were started on postnatal day (PND) 40 (see Fig. 1) and conducted under dim red illumination between 10:00 h and 18:00 h to avoid the transitions between the L-D dark cycle. Food and water were provided *ad libitum*. All animals were acquired and cared for in accordance with the guidelines published in the NIH Guide for the Care and Use of Laboratory Animals. All methods and procedures described below were pre-approved by the Northeastern University Institutional Animal Care and Use Committee under protocol number 23-0407 R. Northeastern University’s animal care and use program and housing facilities are fully accredited by AAALAC, International. The protocols used in this study followed the ARRIVE guidelines for reporting in vivo experiments in animal research [19]. Animals were monitored daily over the duration of the study for general health, food and water consumption. A 15% loss in body weight was set as a humane endpoint.

### Psilocybin preparation and administration

Psilocybin was acquired from the National Institute on Drug Abuse (NIH/NIDA, Bethesda, MD) Drug Supply Program. Animals were randomly divided into two groups: vehicle and PSI. Body weights ranged between 19 and 23 gm. Each mouse received a 100 µl gavage (syringe 2”-curved needle w/ 2 mm ball-tip) for an approximate dose of 3.0 mg/kg of PSI. The dose was chosen based on published data showing PSI given in doses of 2 to 3 mg/kg rapidly affect mouse behavior and 5HT2A signaling [20–22]. Head twitching was observed within minutes of treatment but was not observed by the 4^th^ or 5^th^ treatment. These data were not recorded and quantified. The vehicle group received a comparable volume 0.9% saline. Mice were gavaged every other day for 10 days for a total of five treatments. The time from postnatal day 40–50 is recognized as mid to late adolescence in mice, a period of neuroplasticity particularly in the prefrontal cortex and dopaminergic system affecting emotional regulation and reward processing [23–25].

### Open-field test

A detailed description of the Open Field test in mice appears in previous publications [17, 18] Five weeks post-PSI administration, anxiety and locomotion were assessed using the Open Field test. Animals explored a 1764 cm^2^ Plexiglas arena (divided into 16 zones: 4 center, 12 periphery) for 4 min under dim red light. ANY-Maze software analyzed video recordings for center time, center entries, and locomotion. Sex differences between vehicle and PSI treatments were compared using a two-way ANOVA. Independent Samples t-test (Welch’s test) was used to analyze the combined male and female data.

### Light dark box

The apparatus consisted of two adjoining compartments: a brightly illuminated open area measuring 27 ×27 x 27 cm, illuminated at 1000 lux, and a dark, enclosed chamber of the same dimensions, illuminated at <1 lux) connected by a small opening (7 × 7 cm) that allowed the mice to move freely between the two sections. The testing session lasting 10 min and was videotaped and scored for the latency to first enter the light compartment, total number of transitions between compartments and cumulative time spent in each compartment. Sex differences between vehicle and PSI treatments were compared using a two-way ANOVA. Independent Samples t-test (Welch’s test) was used to analyze male and female data combined. Data was analyzed using GraphPad Prism 10.

### Image set-up and data acquisition

A detailed description of the mouse imaging system is published elsewhere [26]. Measures of brain volume using voxel based morphometry (VBM) and brain microarchitecture using diffusion weighted imaging (DWI) began on PND 85, ca 6 weeks from the first exposure to PSI. These structural data were collected while mice were under light 1% isoflurane anesthesia as previously described [15]. The respiration rate was between 50 and 55 breaths/min. Approximately 9 weeks after PSI exposure, on PND 107, resting state functional connectivity (rsFC) was performed and on PND 176, ca 20 weeks post exposure, odor-induced changes in BOLD activity were conducted. These functional imaging studies were done in fully awake mice following five days of acclimation to the awake imaging procedure.

Imaging sessions were conducted under dim-red illumination using a Bruker Biospec 7.0 T/20-cm USR horizontal magnet (Bruker; Billerica, MA) and a 2 T/m magnetic field gradient insert (ID = 12 cm) capable of a 120-µsec rise time. At the beginning of each imaging session, a high-resolution anatomical data set was collected using the rapid acquisition relaxation enhancement (RARE) pulse sequence (RARE factor 8); (18 slices; 0.75 mm; field of view (FOV) 1.8 cm^2^; data matrix 128 × 128; repetition time (TR) 2.1 sec; echo time (TE) 12.4 msec; Effect TE 48 msec; number of excitations (NEX) 6; 6.5 min acquisition time).

### Voxel based morphometry

A detailed description can be found in Harris-Blum et al. [17]. In brief, a 3D mouse brain atlas with 139 labeled regions (from Ekam Solutions) was used to analyze brain volumes. Volumes for all 139 regions were calculated by counting voxels and multiplying by voxel size. All volumes were normalized by total brain volume to account for size differences between subjects (See Supplementary Data 1 VBM for volumetric data from each subject for all brain areas). The data were evaluated for normality using Shapiro-Wilks test and failed. The data from vehicle and PSI treatments for all subjects for each brain area were normalized to fraction of brain volume and compared using a Wilcoxon matched paired test. To test for sex x treatment interaction data were analyzed using a two-way ANOVA without normality testing. Analyses were performed with GraphPad Prism 10. Analysis were also conducted for sex differences in 13 brain regions e.g., hippocampus, basal ganglia, prefrontal cortex. Each of the 13 brain regions is comprised of different brain areas. Supplementary Data 2. Brain Regions lists all 13 regions and their associated brain areas.

### Diffusion weighted imaging – quantitative anisotropy

The data acquisition, preprocessing, reconstruction and analysis are described in detail in previous publications [27, 28]. In brief, DWI was acquired using spin-echo EPI pulse sequence. Fractional anisotropy (FA) and apparent diffusion coefficient (ADC) maps were generated using MATLAB and MedINRIA software. Brain volumes were registered to a mouse atlas for statistical comparison. The data were non-normal using Shapiro-Wilks test. (See Supplementary Data 3. FA, and Data 4. ADC for data from all subjects). The data from vehicle and PSI treatments for all subjects for each brain area were compared using a Wilcoxon matched paired test. To test for sex x treatment interaction data were analyzed using a two-way ANOVA without normality testing. Analyses were performed with GraphPad Prism 10.

### Resting state functional connectivity

All mice were acclimated to the awake imaging procedure as described previously [29]. The detailed description of the data acquisition, preprocessing, registration, and analysis of rsFC has been previously described [18, 30, 31]. Network analysis was performed in Gephi software using undirected networks from absolute connectivity matrices. Degree centrality was calculated as the sum of connections between each node and all other nodes. The final analysis included six males and four females (*n* = 10) for PSI and six males and five females (*n* = 11) for vehicle treatment. These final sample sizes were underpowered for determining sex differences. The statistical analysis used GraphPad Prism 9.0, with Shapiro-Wilk tests determining normality. Paired t-tests or Wilcoxon signed rank tests (for non-normal data) compared degree centrality between groups.

### Odor-induced BOLD imaging

All mice were acclimated to the awake imaging procedure. A detailed description of the data acquisition, preprocessing, registration, and analysis for BOLD imaging has been previously described [32–34]. Each functional imaging session consisted of uninterrupted data acquisitions (whole brain scans) of 150 scan repetitions or acquisitions for a total elapsed time of 15 min. The control window included the first 50 scan acquisitions (18 slices acquired in each), covering a 5 min baseline. Following the control window, an odor stimulant was introduced through nose cone attached to a pump for a flow rate of 5.0 ml/min. The odor stimulus continued for the duration of the scanning session. The first odor was benzaldehyde the smell of almond. Both vehicle and PSI treated mice were exposed to almond, a rewarding stimulus. Two weeks later mice were exposed to TMT (trimethylthiazoline) a compound found in fox feces and urine that produces a predator odor that causes innate fear responses in rodents [35]. Since all mice were odor naïve to both stimuli we chose the almond odor first to avoid any association with a highly negative stimulus of TMT during the second scanning session.

Statistical analyses were performed on a voxel-by-voxel basis across the entire brain volume (approximately 36,000 voxels) within each subject’s native coordinate space. Prior to hypothesis testing, we implemented a conservative signal change threshold to exclude voxels with minimal physiological relevance. Only voxels demonstrating BOLD signal changes exceeding ±1% from baseline during the odor period were retained for statistical analysis. This preprocessing step was designed to eliminate noise-related fluctuations and minor physiological drift while preserving voxels with potentially meaningful neural responses. For each qualifying voxel, we conducted one-sample t-tests against the null hypothesis of zero change from baseline. Voxel comparisons were made between baseline (1–50 image acquisitions) and the initial odor presentation (51–100 image acquisitions). Statistical testing employed a two-tailed distribution with heteroscedastic variance assumptions and a significance threshold of *p* < 0.05 (95% confidence interval). To address the multiple testing, we implemented false discovery rate (FDR) correction using the Benjamini-Hochberg procedure across all voxels. The FDR was controlled at Q = 0.05 See Supplementary Data 5 and 6 for Reward and Fear analysis and tables, respectively.

### Prefrontal cortex isolation, protein and statistical analysis

Approximately 12 weeks post PSI exposure and following odor imaging, mice were euthanized and the brains frozen on dry ice and stored at -80 °C. From frozen brains, the olfactory bulbs were removed and ~1 cm was dissected out that included the part of the cerebral cortex, prelimbic cortex and prefrontal cortex. Samples were lysed in RIPA buffer with protease inhibitor through Douce homogenizer (20 strokes small) followed by sonification (20 s) on ice.

#### Western blot methodology

Western blot analysis was performed using SDS-PAGE system. Protein samples were separated on precast 4–20% ExpressPlus PAGE gels (GenScript, M41212) for 1 h 15 min at 140 volts using a Invitrogen PowerEase Touch system. Proteins were transferred onto PVDF membranes (Miliporesigma) using the eBlot L1 Transfer System (Genescript). Membranes were then blocked in 5% milk/TBST for 1 h at 24 °C before incubation with primary antibodies for overnight (12–16 h) at 4 °C. Membranes were then washed 3 times (10 min each) with TBST and probed for secondary antibodies for 1 h at room temperature. Membranes were then washed again 3 times (10 min each) with TBST. The bands were visualized using enhanced chemiluminescence (ECL) detection kit (Thermo Fisher Scientific). Anti-beta tubulin was used as a loading control.

Standard Western protocol as previously described [36]. Repressor Element 1 Silencing Transcription factor (REST, PTG 22242-1), Regulator of Calcineurin 1 (RCAN1, PTG 14869-1), protein phosphatase 1 regulatory inhibitor subunit 1B (PPP1R1B,DARPP-32, CST 2306), aquaporin (AQP4, CST 59678), acetylated lysine (CST 944), all normalized to tubulin (E7, developed by Drs. M. McCutcheon and S. Carroll) was obtained from the Developmental Studies Hybridoma Bank, created by the NICHD of the NIH and maintained at The University of Iowa, Department of Biology, Iowa City, IA 52242).

#### Normalization method

Band pixels were measured using UN-SCAN-IT software (https://www.silkscientific.com/gel-analysis.htm). Individual band pixel was then normalized against the respective tubulin band pixel. Each lane represents one biological replicate. Ratios of specific proteins and tubulin were displayed as scattered plot with mean ± SD. All Western data were analyzed using a two-way ANOVA with Sidak’s correction as mean±SD. *p < 0.05, **p < 0.002, ***p < 0.0002. All biological replicates with male/vehicle(*n* = 5), male/PSI (*n* = 4), female/vehicle (*n* = 3), and female/PSI (*n* = 6).

## Results

### Experimental timeline and behavior

Shown in Fig. 1 is the experimental timeline given as postnatal days (PND) and the behavioral data. Oral gavage of mice with PSI or vehicle started on PND 40. Four weeks post treatment mice were evaluated in the Light/Dark Box and Open Field. A two-way ANOVA showed no significant differences for each dependent measure in the Light/Dark box (*p* > 0.05) (Fig. 1b). There was no significant main effect for sex by treatment for time in the light (F = 2.089, *p* = 0.162) or time in dark (F = 2.072, *p* = 0.164). When the data between sexes were collapsed (vehicle n = 13, PSI *n* = 14) there was no significant difference between groups for the light box entries (*p* = 0.6020) or dark box entries (*p* = 0.567) using an independent sample t-test. However, in the Open Field (Fig. 1c) there was a significant interaction between sex by treatment for Center Crossing (F = 10.780, *p* = 0.003), a trend toward significance for Total Distance traveled (F = 3.579, *p* = 0.070) while Average Speed (F = 3.063, *p* = 0.093) was insignificant. A Tukey’s multiple comparison’s post hoc test showed that the vehicle treated females were significantly greater in all measures as compared to PSI treated male and female mice and vehicle treated males. However, PSI treatment significantly decreased female behavior as compared to vehicle treated females. When the data between sexes were collapse (vehicle *n* = 14, PSI *n* = 14) there were significant differences for the Total Distance traveled (*p* = 0.0287), Average Speed (*p* = 0.042) and Center Crossings (trending *p* = 0.058).

### Voxel based morphometry

**Fig. 2:**
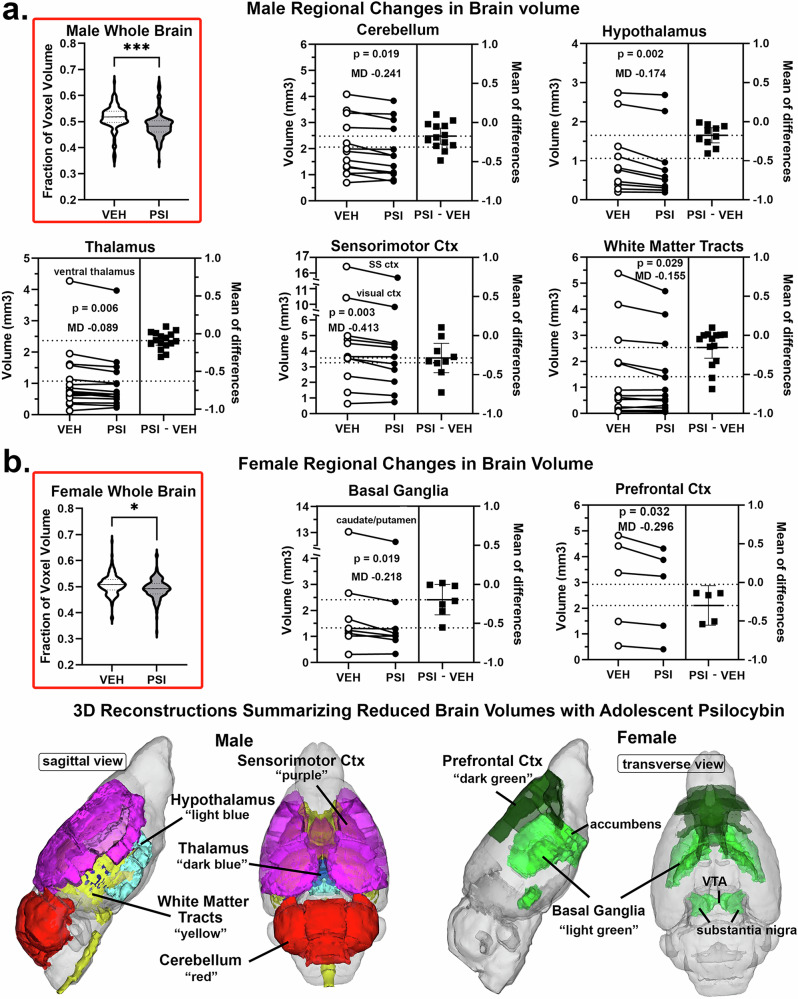
Changes in voxel based morphometry. Shown are the developmental changes in brain volumes for males **a** and females **b** following early exposure to psilocybin (PSI). Highlighted in red are the difference in whole brain volume for males and females shown as violin plots (median and range) for all 139 brain areas after normalization to correct for different whole brain volumes for each subject and then as a fraction of the voxel volume for each brain area to normalize for the large differences in voxel numbers between brain areas. The estimation plots show the difference between vehicle (open circle) and PSI (closed circle) in the individual brain areas and there mean difference (closed squares). Included in each brain region is the *p* value and the average mean difference. Below are 3D color coded reconstructions summarizing the significantly different brain regions and their difference between sexes. * *p* < 0.05, *** *p* < 0.001.

A whole brain two-way ANOVA for VBM looking for a sex by treatment interaction was not significant (F = 0.676, *p* = 0.421). Even without a significant interaction, males showed 2.6× larger volumetric reductions than females. This analysis was also performed for the 13 brain regions and again there was no interaction. Analysis of sex by treatment interaction for the 139 individual brain areas showed 10 brain areas with a significant interaction (uncorrected *p* < 0.05), with the strongest effects in thalamic areas. Due to the small sample size no areas survived FDR correction. Consequently, comparisons between vehicle and PSI treatments were confined to each sex (Fig. 2). In both males and females there was a significant decrease in whole brain volume with PSI as shown as violin plots framed in red. These data are shown as a fraction of the voxel volume to normalize for the large differences in voxel numbers between brain areas. PSI treatment in adolescence significantly decreases the whole brain volume in males (*p* < 0.001) and females (*p* < 0.05). Figure 2a shows the regional changes in brain volume (mm^3^) for vehicle (open circle) and PSI (closed circle) treated males for each brain area localized to that brain region. For example, the cerebellum has 11 brain areas (see Supplementary Data 2 Brain Regions for list of individual areas) each shown as connected circles. The mean of differences (MD) between each is shown to the right as a filled square. There was a significant decrease in cerebellum brain volume (paired -t test, *p* < 0.019) with a MD of -0.241 mm^3^. Of the thirteen brain regions identified in the mouse brain atlas the cerebellum, hypothalamus, thalamus, sensorimotor cortex, and white matter tracts present with a significant decrease in brain volume for males. The organization of these brain regions are shown a color-coded 3D reconstructions below. The regional differences in brain volumes for females is shown in Fig. 1b. Only the basal ganglia (*p* < 0.019, MD -0.218 mm^3^) and the prelimbic cortex (*p* < 0.031, MD -0.255) are significantly reduced with PSI treatment. The fact that there is no overlap between males and females in the affected brain regions suggests PSI has sex dependent site-specific effects on brain structure.

### Diffusion weighted imaging

A whole brain two-way ANOVA for FA and ADC values testing for a sex x treatment interaction was not significant (FA: F = 0.662, *p* = 0.424; ADC F = 2.672, *p* = 0.116). This analysis was also performed for the 13 brain regions and again there was no interaction. Analysis of sex x treatment interaction for the 139 individual brain areas showed three brain areas for FA and five for ADC with a significant interaction (uncorrected *p* < 0.05), but no areas survived FDR correction. Consequently, comparisons between vehicle and PSI treatments were confined to each sex. The table of FA values in Supplementary Data 3. FA shows the significant changes in measures of FA between mice (male and female combined) treated with vehicle or PSI during adolescence for 139 brain areas. In all cases, 48/139 brain areas showed PSI increases FA. In contrast, the table of ADC values in Supplementary Data 4. ADC lists only 11/139 brains areas that showed significant changes in ADC values. In all cases, PSI treatment increased ADC values.

**Fig. 3:**
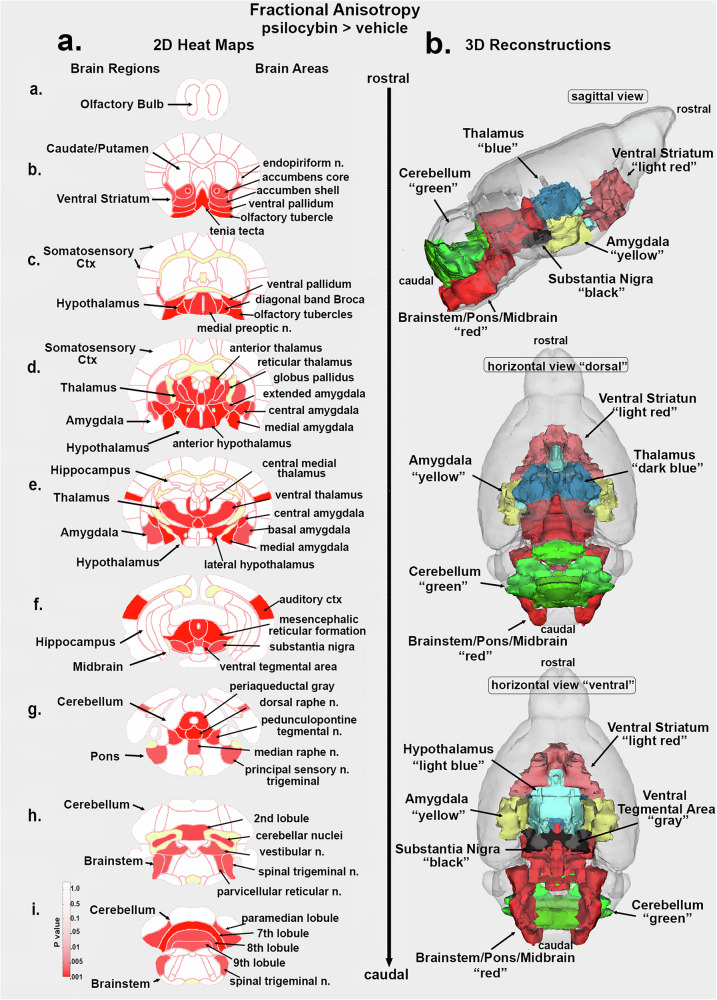
Mapping fractional anisotropy differences. Shown in Fig. 3**a**. are 2D images of statistical heat maps highlighting the location of brain regions that were significantly different in fractional anisotropy (FA) values between vehicle and PSI. Areas in red denote a significant increase in FA values over vehicle for PSI treatment. The yellow identifies white matter tracts. The 3D color coded reconstructions to the right in Fig. 3**b**. summarize these regional difference in measures of FA.

The location of many of the significantly affected brains areas reported in the FA table in Supplementary Excel 3 are shown in the 2D maps and summarized in the 3D reconstructions in Fig. 3. These brain areas are presented as statistical 2D heat maps. The coronal sections are labeled (a.) through (i.) and arranged from rostral (top) to caudal (bottom) with major brain regions shown to the left and specific brain areas listed to the right. Note the absence of any changes in the regions of the olfactory bulb (a.) caudate/putamen (b.) and somatosensory cortex (b.- d.) While the ventral striatum (accumbens, ventral pallidum, and olfactory tubercles) (b., c.) with dopaminergic connections from the ventral tegmental area (f.) are affected by early exposure to PSI. Many areas of the thalamus (e.g., reticular, anterior and ventral) are affected (d., e.) as are the amygdala (e.g., central, medial and basal) (d., e.) and hypothalamus (e.g., anterior and lateral) (d., e.). The substantia nigra (f.) periaqueductal gray and raphe (f. g.) and several areas in the cerebellum (g. – i.) were significantly affected. Interestingly, the hippocampus remained unaffected by early exposure to PSI (e., f.), Given the substantial number of affected areas, the locations are better summarized as brain regions shown as color-coded 3D reconstructions to the right of Fig. 3.

**Fig. 4:**
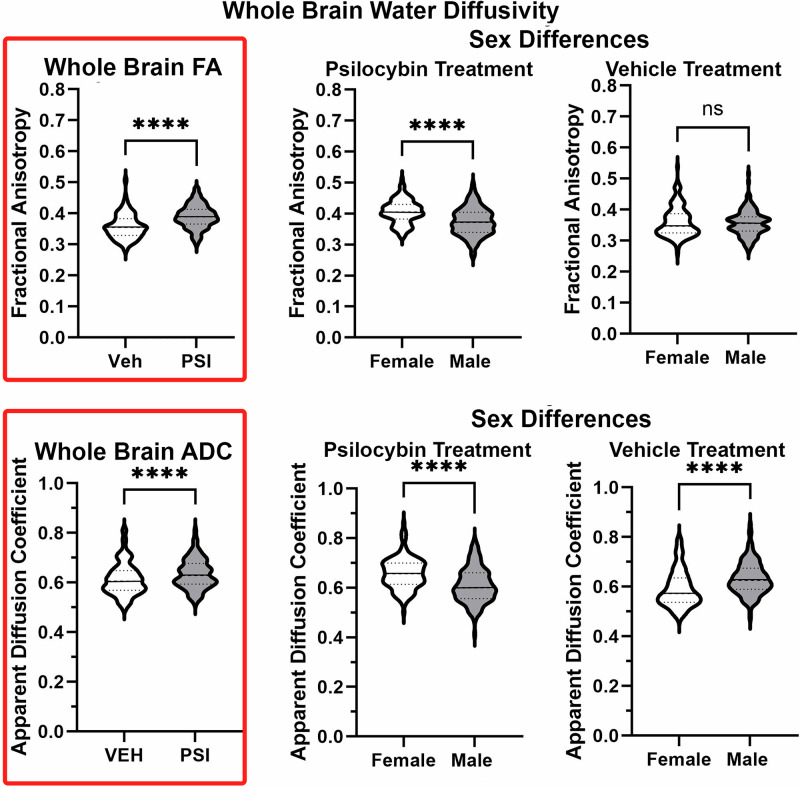
Whole brain water diffusivity. Shown highlighted in red are violin plots (median and range) for measures of fractional anisotropy (FA) and apparent diffusion coefficient (ADC) for all 139 brain areas. For both measures of water diffusivity PSI treated mice show higher values. When parsed into sexes females have significantly higher measures of both FA and ADC as compared to males exposed to PSI. *** *p* < 0.001, ns non-significant.

Figure 4 shows the significant differences for whole brain between vehicle and PSI treatments for measures of FA and ADC. The violin plots (highlighted in red) for males and females combined show early PSI treatment significantly increases global FA and ADC measures (*p* < 0.0001). While the change in whole brain FA and ADC values increase with PSI treatment males present with significantly lower values (*p* < 0.0001) than females with PSI. Interestingly, with vehicle treatment there is no sex difference for FA, but males are significantly greater (*p* < 0.0001) than females for ADC.

### Resting state functional connectivity

**Fig. 5:**
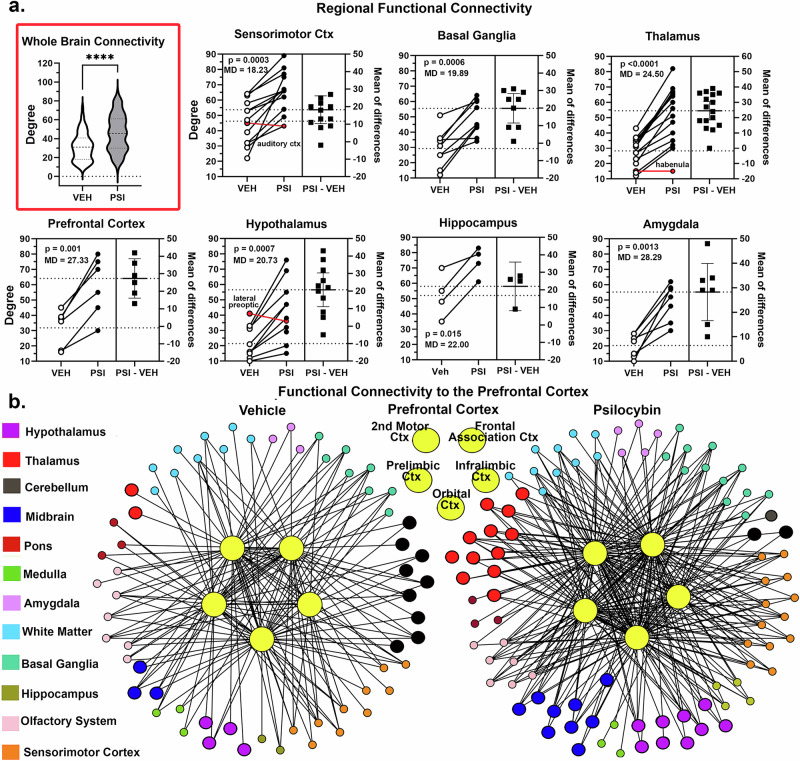
Functional connectivity. Shown in Fig. 5**a**. highlighted in red are the connections between nodes i.e., degrees (a.) for the whole brain comprised of 139 brain areas. To the right are estimation plots for different brain regions. The estimation plots show the difference between vehicle (open circle) and PSI (closed circle) in the individual brain areas and there mean difference (closed squares). Included in each brain region is the *p* value and the average mean difference. Below in Fig. 5**b**. are radial maps showing the connections (lines) between the five brain areas that comprise the prefrontal cortex (large yellow dots) and surrounded brain areas color coded for their brain region. The enlarged circles highlight the brain regions most affected by early exposure to psilocybin.

The change in degrees (connections) for the whole brain, male and females combined, between vehicle and PSI treatments are shown in Fig. 5a as violin plots (highlighted in red). Early treatment with PSI significantly increases functional connectivity as compared to vehicle (*p* < 0.0001). The PSI global network density was 0.333, average degree 45.681, and average path length 1.758. The vehicle global network density was 0.226, average degree 31.0, and average path length 1.895. These changes are region specific. Shown are seven brain regions that present with a significant increase in the number of degrees with PSI treatment (closed circles) as compared to vehicle (open circles) for each brain area in the region. Each regional plot shows the significance (*p* value) and median difference (MD) and the mean of differences (closed box). All brain areas in each brain region show a large increase in degrees with PSI with only a few exceptions. In the sensorimotor cortex, thalamus, and hypothalamus, there is an area in each (red bar) that shows a decrease. Given the importance of the prefrontal cortex in adolescent development the organization of the functional connections to this region are show in Fig. 5b below. The five prefrontal areas e.g., secondary motor cortex, frontal association cortex, prelimbic and infralimbic cortices and the orbital cortex as shown as yellow circles. The degrees (connecting lines) and nodes (circles) associated with the prefrontal cortex are arranged in a surrounding radial pattern, color coded for different brain regions. There are 62 nodes and 175 degrees with vehicle treatment and 90 nodes and 310 degrees with PSI. The brain regions most affects by PI are highlighted by enlarged circles and include the hypothalamus (purple), thalamus (red), cerebellum (black) and midbrain (dark blue). The thalamus goes from 2 nodes to 11, the hypothalamus from 4 to 9 and the midbrain from 3 to 9. Interestingly, the cerebellum decreased with PSI treatment, going from 9 to 2.

### Odor-induced BOLD imaging

**Fig. 6:**
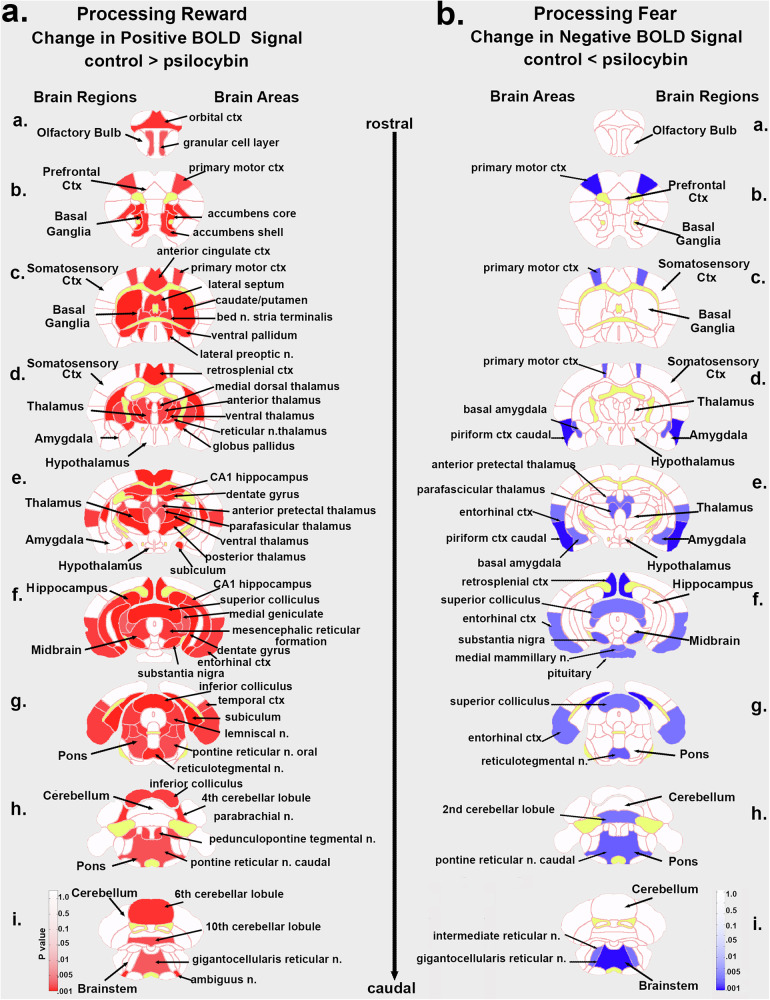
Sensory perception. The heat maps (Fig. 6**a**, **b**.) highlight the brain areas that were significantly different between control and PSI mice following exposure to the rewarding smell of benzaldehyde (almond) or the aversive fearful smell of TMT (fox). When processing reward (Fig. 6**a**.) the changes were predominantly positive BOLD (red) where controls were significantly greater than PSI mice. When processing fear (Fig. 6**b**.) the significant changes were all negative BOLD (blue) where controls were less than PSI than PSI mice.

Mice were acclimated to awake imaging procedure. During the scanning session vehicle and PSI treated mice were presented with the smell of almond (benzaldehyde) and two weeks later the smell of fox (TMT). Give the reduced number of mice in each experimental group (female vehicle *n* = 4, male vehicle *n* = 6, female PSI *n* = 6, and male PSI *n* = 6) and the variability in awake imaging the study was underpowered to perform analysis for a sex x treatment interaction. Hence, males and females were combined for each treatment. There was a significant difference between vehicle and PSI treatment in response to almond with 50/139 brain areas showing higher positive BOLD signal in veh over PSI (see positive BOLD in Supplement Data 5. Reward) suggesting mice with early exposure to PSI are less sensitive to rewarding stimuli. Similarly, when exposed to a fearful stimulus like fox scent, PSI mice showed a significantly higher negative BOLD as compared to vehicle in 27/139 brain areas (see negative BOLD in Supplement Data 6. Fear) suggesting a reduction in brain activity to aversive stimuli. The brain areas that show a difference in positive BOLD signal toward almond odor and the negative BOLD signal to fox scent are presented as heat maps in Fig. 6.

### Protein biomarkers of neuroplasticity

**Fig. 7:**
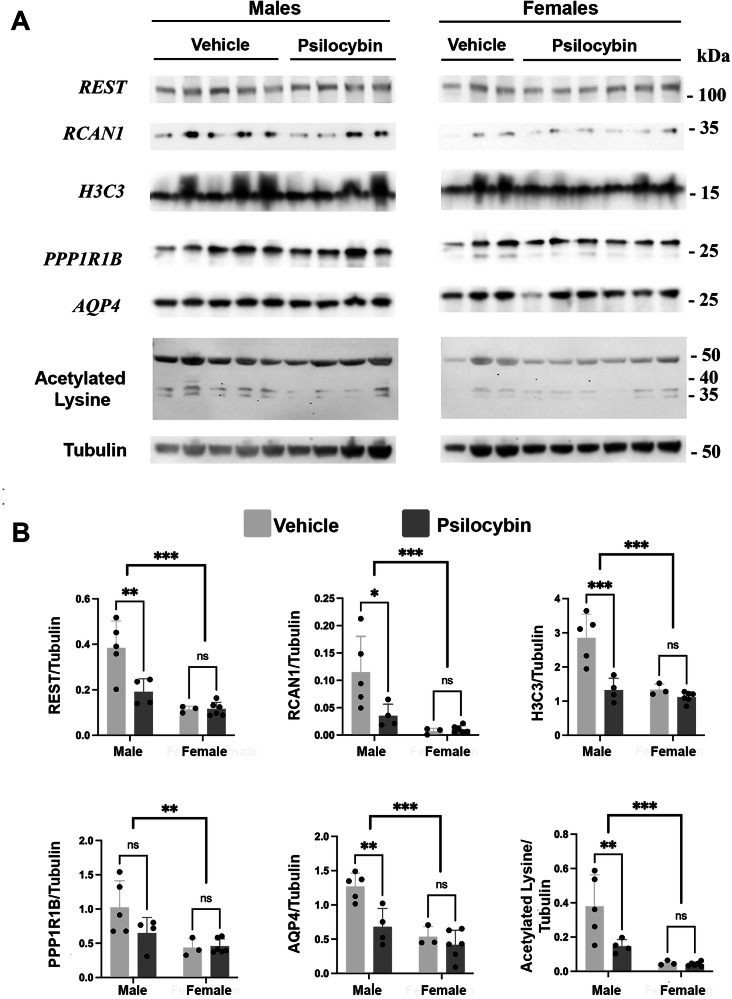
Protain associated epigenetic changes. **A** Western blots probed for REST, RCAN1, H3C3, PPP1R1B, AQP4 and beta-tubulin. **B** Quantification of the REST, RCAN1, H3C3, PPP1R1B, AQP4, and acetylated lysine normalized to beta-tubulin from each corresponding biological sample. Data were analyzed using a two-way ANOVA with Sidak’s correction and presented with error bars representing ± SD. *P < 0.05, **P < 0.002, ***P < 0.0002. Each lane represents a biological replicate with male/vehicle (*n* = 5), male/PSI (*n* = 4), female/vehicle (*n* = 3), and female/PSI (*n* = 6). RIPA soluble cerebral cortex, prelimbic cortex and prefrontal cortex. Sex x treatment interaction REST (F = 7.879, *p* = 0.014); RACN1 (F = 5.338, *p* = 0.036); H3C3 (F = 10.29, *p* = 0.0063); AQP4 (F = 5.181, *p* = 0.0391); Acetylated Lysine (F = 5.424, *p* = 0.0354); PPP1R1B (F = 2.716, *p* = 0.1216).

To investigate changes in proteins associated with neuroplasticity PSI treated adolescents we examined the expression of several proteins that participate in epigenetic remodeling. We found most proteins were downregulated in male mice and when combining male and female mice regardless of treatment there were global sex-dependent changes (Fig. 7). REST functions as a neuron-restrictive silencer factor and serves as a key regulator of neurogenesis and neural differentiation and overall gene expression [37]. REST assembles to target gene histone deacetylases 1 and 2, histone methyl-transferases to promote epigenetic remodeling and gene silencing and are implicated in the pathogenesis of many neurodegenerative diseases [38]. There was a significant reduction (*p* = 0.0024) in REST protein expression upon PSI treatment compared to vehicle treated male mice (vehicle, *n* = 5; PSI, *n* = 4). In addition, there was a significant decrease (*p* = 0.0123) in the protein expression of Regulator of Calcineurin 1 (RCAN1) gene in PSI treated male mice compared to the vehicle treated group. RCAN1 is implicated in epigenetic regulation of Alzheimer’s and kidney diseases [39, 40]. We next examined the H3 variant of histone (H3C3) protein expression in both males and females and noticed a significant reduction (*p* = 0.0002) in the PSI treated male mice. H3C3 is critically involved in the epigenetic regulation of gene expression required for maturation, memory and learning [41]. In addition, PPP1R1B or DARPP-32 *c*AMP-regulated phosphoprotein while not associated with neuroplasticity, is involved in muscle development and plays a role in working memory [42, 43]. PPP1R1B showed no significant change in protein levels in PSI treated animals; however, there is a significant sex-difference between male and female mice (*p* = 0.0059). AQP4 is a water channel protein primarily expressed in astrocytes and ependymal cells and could serve as a marker for gliogenesis [44]. Interestingly, AQP4 level is significantly reduced (*p* = 0.002) in PSI treated male mice. Global acetylated lysine is a key mechanism to regulate chromatin structure and aberrant acetylation has been implicated in many neurodevelopmental and degenerative diseases [45] and PSI significantly reduced (*p* = 0.0074) the level of acetylated lysine in the male mice. Surprisingly, none of these markers showed any notable changes between vehicle and PSI treated groups in the female mice. Interestingly, when comparing male and female mice there was a significant sex by treatment interaction for all protein markers except PPP1R1B (see F values and significance in legend).

## Discussion

This study provides the first comprehensive neuroimaging assessment of long-term developmental consequences following PSI exposure during adolescence. Our findings reveal profound and sexually dimorphic alterations in behavior, brain structure, and brain function and epigenetics that persist into adulthood, with males showing more pronounced structural and molecular changes than females.

### Changes in behavior

The most striking behavioral finding was the pronounced sex-specific suppression of locomotor activity in females following adolescent PSI exposure, which eliminated the heightened exploratory behavior observed in vehicle treated female mice. This female-selective behavioral vulnerability occurred despite males exhibiting more extensive structural and molecular alterations, revealing a dissociation between neurobiological changes and behavioral outcomes. This sex-dependent response contrasts with acute PSI studies in adult rodents, which report minimal effects on locomotion at therapeutic doses [20, 46], and highlights critical distinctions between acute adult responses and persistent developmental consequences. The enhanced female sensitivity aligns with emerging evidence that females exhibit greater PSI-induced responses [47], including significantly higher head-twitch frequencies across multiple compounds [48, 49], likely reflecting estrogen-mediated modulation of 5-HT_2A_ receptor expression and signaling [50].

### Structural brain changes

The observed whole-brain volume reductions following early PSI exposure represent a novel finding in the developmental psychedelic literature. To our knowledge, this is the first study to use multimodal MRI to characterize lasting structural and microstructural changes following adolescent PSI exposure. While a recent study from our group examined structural consequences of adolescent LSD exposure using a similar methodology [17], no volumetric changes were observed despite pronounced alterations in diffusion anisotropy and functional connectivity. The discrepancy between LSD and PSI effects may reflect differences in receptor binding profiles, duration of action, or developmental timing of exposure. The regional pattern of these changes—predominantly affecting cerebellum, hypothalamus, thalamus, sensorimotor cortex, and white matter in males, while being more limited in females—suggests sexually dimorphic responses to developmental PSI exposure. These volume reductions do not appear to reflect neurodegeneration, as they occurred without behavioral deficits and were accompanied by enhanced functional connectivity. The concurrent elevation in both FA and ADC values are counter intuitive. Typically, increased ADC suggests reduced tissue density or cellular organization, while elevated FA indicates enhanced directional water diffusion, often associated with improved white matter integrity [51]. The co-occurrence of both changes, particularly the widespread FA increases across 48 brain regions, may reflect a developmental reorganization process where reduced overall tissue density is accompanied by enhanced directional organization of remaining neural elements. This pattern is consistent with accelerated synaptic pruning, a normal developmental process that eliminates redundant connections while strengthening functionally important pathways [52]. Previous work in our lab by Coleman et al. [15] found similar FA elevations in female mice exposed to adolescent cannabis, which we interpreted as enhanced prefrontal organization. Our findings extend this observation to PSI and demonstrate that such changes can occur alongside volume reductions, suggesting a complex reorganization rather than simple loss of tissue. Recent human studies by Siegel et al. using functional mapping demonstrated that acute PSI in adults produces massive disruption of functional connectivity lasting up to 3 weeks, with persistent hippocampal-default mode network desynchronization [53]. However, these studies did not assess volumetric or diffusion changes, highlighting the importance of our developmental multimodal approach. The persistence of our observed structural alterations months after exposure suggests that adolescent exposure may produce more enduring architectural changes than acute adult administration.

The counterintuitive co-occurrence of increased FA and ADC has not been previously reported in psychedelic research, though recent adult studies provide mechanistic context. Shao et al. demonstrated using two-photon microscopy that adult mice given PSI show rapid (within 24 h) and persistent (1 month) increases in dendritic spine density (~10%) and size in frontal cortex, driven by elevated spine formation rates [54]. Indeed, there is accumulating evidence that PSI increases dendritic complexity, synaptic protein expression, and neurogenesis markers in adult rodents. However, these cellular-level increases occurred in mature animals and were not assessed with in vivo MRI. Our findings of reduced volume alongside elevated FA/ADC in adolescent mice suggest that developmental exposure may produce a distinct pattern of structural reorganization, where enhanced directional organization (FA) and increased water diffusion (ADC) co-occur with overall tissue volume reduction. This could reflect accelerated or exaggerated synaptic pruning during a critical developmental window, where the normal refinement process is amplified by psychedelic-induced plasticity signals.

### Enhanced functional connectivity

The sustained increase in global functional connectivity following early PSI exposure aligns with Carhart-Harris’s entropic brain hypothesis, which proposes that psychedelics enhance network integration while reducing segregation between brain regions [55]. However, our developmental findings reveal important distinctions from acute adult studies. While acute PSI typically produces transient desynchronization in association networks, particularly the default mode network [56], our developmental exposure resulted in persistent hyperconnectivity that was particularly pronounced in cortico-striato-thalamo-cortical circuits. As noted above, Siegel et al. found that acute PSI caused desynchronization in the default mode network and its connections to thalamus, basal ganglia, hippocampus, and cerebellum, with most connectivity normalizing by three weeks except for persistent default mode network-hippocampus desynchronization [53]. Our developmental study shows sustained increases in global connectivity and whole-brain entropy following early PSI exposure, supporting the entropic brain hypothesis in a developmental context.

Acute PSI consistently reduces intra-network connectivity in association cortices, including default mode and salience networks in healthy participants [57, 58]. Preller et al. documented temporal dynamics showing initial occipital hyperconnectivity at 20 min, followed by association network hypoconnectivity and enhanced sensory system connectivity at 40–70 min [58]. Our findings align with these observations of PSI-induced global connectivity increases across sensory networks, particularly in cortico-striato-thalamo-cortical circuits involved in sensory filtering. The pattern of prefrontal cortex hyperconnectivity, showing enhanced connections to hypothalamus, midbrain, and thalamus while displaying reduced connectivity to cerebellum, suggests a fundamental reorganization of executive control networks. This connectivity profile may represent an adaptive response to early PSI exposure, potentially reflecting enhanced top-down regulatory capacity over subcortical regions involved in motivation, arousal, and homeostasis [59].

### Altered sensory processing

Perhaps the most functionally significant finding was the altered neural response to evolutionarily relevant odors in PSI-exposed mice. The reduced BOLD activation to benzaldehyde (almond scent), an innately rewarding stimulus that typically activates motivation and reward circuits [60–62], and the enhanced negative BOLD response to trimethylthiazoline (fox scent), an innately aversive stimulus, suggest a global change of sensory-affective processing. Febo et al., pretreated lactating dams with the neuropeptide oxytocin and then challenged them with TMT during the MRI scanning and reported a significant increase in negative BOLD as reported here [35]. The oxytocin-primed, TMT-induced negative BOLD was interpreted as a decrease in neuronal activity causing a reduction in freezing behavior, i.e., mothers were less fearful. These findings are particularly significant in light of recent evidence demonstrating that psychedelics fundamentally alter the integration of sensory perception with reward and fear processing networks. A study by Nardou and colleagues demonstrated that psychedelics could reopen the critical period in development involved in social reward learning by restoration of oxytocin-mediated long-term depression in the nucleus accumbens, a key node in reward processing [63]. This mechanism may help explain the reduced sensitivity to rewarding and fearful stimuli observed in our adolescent-exposed mice, as the nucleus accumbens is critically involved in processing the hedonic value of sensory cues, including olfactory stimuli.

The hypo-responsiveness is particularly noteworthy given the enhanced structural connectivity observed in the same animals. The increased anatomical connections alongside reduced functional responsiveness may reflect a compensatory mechanism where enhanced baseline connectivity reduces the system’s dynamic range for stimulus-evoked responses [64]. Alternatively, this pattern might represent a form of sensory gating enhancement, where the brain becomes more selective in its responses to environmental stimuli [65]. From an evolutionary perspective, this altered sensory processing could have significant adaptive implications. The reduced responsiveness to both rewarding and aversive stimuli might influence risk assessment, reward seeking, and environmental exploration behaviors in ways that could affect survival and reproduction. Future studies should examine whether these changes extend to other sensory modalities and whether they translate to altered behavioral responses to natural stimuli.

### Molecular mechanisms: Sex-specific epigenetic remodeling

The male-specific downregulation of multiple neuroplasticity-related proteins in the prefrontal cortex provides crucial mechanistic insights into the observed structural and functional changes. The coordinated reduction of REST, RCAN1, histone H3, AQP4, and acetylated lysine suggests a comprehensive epigenetic remodeling process that may underlie the persistent developmental alterations. REST (RE1-Silencing Transcription factor) typically functions as a transcriptional repressor that maintains neuronal gene silencing in non-neuronal cells and regulates neuronal maturation [37, 38]. Its downregulation months after PSI treatment could reflect a compensatory mechanism to maintain neuronal gene expression in the face of the enhanced connectivity observed in the prefrontal cortex. The reduction in RCAN1 (Regulator of Calcineurin 1) is particularly intriguing given calcineurin’s central role in synaptic plasticity and long-term depression [66, 67]. RCAN1 normally acts as a brake on calcineurin signaling, so its downregulation could facilitate ongoing synaptic refinement processes associated with the observed hyperconnectivity. The concurrent reductions in histone H3 [41] and acetylated lysine [45] indicate broader epigenetic remodeling that may establish lasting changes in gene expression patterns [68, 69]. This epigenetic signature could represent a form of “developmental memory” that maintains the altered brain organization established during the initial PSI exposure period.

Multiple studies show PSI rapidly upregulates plasticity-related genes, synaptogenesis and dendritic growth in the prefrontal cortex within hours of administration [54, 70]. Psilocin given to human cortical neurons upregulates proteins and genes associated with synaptogenesis, synaptic transmission and neuronal complexity [71]. Rats exposed to LSD present with enhanced gene expression of epigenetic proteins in the prefrontal cortex that lasts for months [72]. Shao et al. demonstrated that single-dose PSI increases dendritic spine density in prefrontal cortex, with more pronounced effects in females [54]. In our study the absence of these molecular changes in females, despite similar functional connectivity alterations, suggests fundamentally different mechanisms underlying PSI’s effects in males versus females. This sexual dimorphism may reflect differences in baseline epigenetic regulation, hormonal influences during adolescence [73], or sex-specific responses to 5-HT_2A_ receptor activation [50, 74].

### Clinical and public health implications

The persistence of these neurobiological changes into adulthood, occurring in the absence of overt behavioral abnormalities, raises important questions about the long-term consequences of adolescent psychedelic use. While our findings do not demonstrate clear adverse outcomes, the altered sensory processing and extensive brain reorganization suggest that developmental PSI exposure may have subtle but lasting effects on how individuals perceive and respond to their environment [75]. The pronounced sexual dimorphism in responses, with males showing more extensive structural changes and unique molecular alterations, has important implications for understanding risk factors and potential therapeutic applications. These findings suggest that sex should be a critical consideration in both research on developmental psychedelic exposure and in clinical applications involving adolescent populations. Given the increasing prevalence of adolescent psychedelic use reported in recent epidemiological studies [8, 9], these findings have immediate relevance for drug policy and harm reduction strategies. The subtle but persistent nature of the observed changes suggests that conventional behavioral assessments may be insufficient for detecting the full scope of developmental psychedelic effects [76].

### Limitations and future directions

Several limitations should be acknowledged. Our study used a single dose regimen (3.0 mg/kg every other day for five exposures) and cannot address dose-dependent effects or different exposure patterns [77]. The mouse model, while valuable for mechanistic insights, may not fully capture the complexity of human adolescent brain development or psychedelic responses [25]. Additionally, our behavioral assessments were limited to anxiety-related measures and did not comprehensively evaluate cognitive function, social behavior, or other domains that might be affected by the observed brain changes. Future studies should examine dose-response relationships, investigate whether these changes are reversible, and assess functional consequences across a broader range of behavioral domains [78]. Long-term longitudinal studies tracking animals into advanced age would be valuable for understanding whether early PSI exposure affects aging-related brain changes or disease susceptibility. Finally, the molecular findings in males warrant particular attention in future research. Investigating the temporal dynamics of these changes in protein associated neuroplasticity and their relationship to the structural and functional alterations could provide crucial insights into the mechanisms underlying PSI’s developmental effects.

## Conclusion

This study provides compelling evidence that PSI exposure during adolescence produces lasting, sexually dimorphic alterations in brain structure, connectivity, and function. The combination of reduced brain volumes, altered tissue microarchitecture, enhanced functional connectivity, dampened sensory responses, and male-specific molecular changes demonstrates that the adolescent brain’s heightened plasticity makes it particularly vulnerable to psychedelic-induced reorganization. These findings have important implications for understanding the developmental neurobiology of psychedelics and informing evidence-based policies regarding adolescent psychedelic use.

## Supplementary information

VBM Values

Brain Regions

FA Values

ADC Values

Reward

Fear

## Data Availability

All data voxel based morphometry and diffusion weighted imaging data generated or analyzed during this study are included in this published article [and its supplementary information files]. The functional connectivity data are available through Dryadz 10.5061/dryad.9kd51c5s9.
